# Beyond Melanoma: Hutchinson’s Sign in Subungual Melanocytic Activation – A Case Report

**DOI:** 10.1159/cde/abvag017

**Published:** 2026-09-07

**Authors:** Fatimah J. Al Muqrrab, Ahmed Alhumidi, Mohammed Al-Haddab

**Affiliations:** a Department of Dermatology, College of Medicine, King Saud University, Riyadh, Saudi Arabia; b Department of Dermatology, College of Medicine, Imam Abdulrahman Bin Faisal University, Dammam, Saudi Arabia; c Department of Pathology, College of Medicine, King Saud University, Riyadh, Saudi Arabia

**Keywords:** Hutchinson’s sign, Subungual melanosis, Longitudinal melanonychia, Dermoscopy, Biopsy, Nail pigmentation

## Abstract

**Introduction:**

Hutchinson’s sign, defined as periungual pigmentation extending into the surrounding nail folds, is conventionally considered a hallmark of subungual melanoma. However, several benign nail disorders may present with Hutchinson’s sign, creating a diagnostic challenge for clinicians.

**Case Presentation:**

We report the case of a 33-year-old Saudi man who developed a progressive brown-black longitudinal band on his left little fingernail accompanied by pigmentation extending into the eponychium. Dermoscopy revealed irregularly spaced parallel lines with brush-like extensions in the proximal nail fold. Given the clinical suspicion of nail melanoma, a tangential nail matrix biopsy was performed. Histopathology demonstrated increased basal melanin deposition with cytologically bland melanocytes, which is consistent with subungual melanocytic activation. The patient was followed for 6 months. The nail completely regrew with no dystrophy, and the band had recurred.

**Conclusion:**

This case highlights that Hutchinson’s sign is not pathognomonic for melanoma and can occur in benign entities such as subungual melanosis. Careful integration of clinical, dermoscopic, and histopathological findings is essential for accurate diagnosis, avoiding unnecessary aggressive interventions while ensuring patient safety.

## Introduction

Hutchinson’s sign, defined as periungual extension of nail pigmentation [1], has long been regarded as a key clinical indication of radial extension of the subungual melanoma [1–3]. While its presence often raises suspicion of malignancy, a range of benign conditions, including melanocytic nevi, ethnic pigmentation, trauma, infections, and drug-induced changes, can mimic this finding [4]. Recognizing these non-melanoma variants is essential to avoid misdiagnosis and unnecessary invasive interventions. We present a rare adult case of benign melanonychia displaying a positive Hutchinson’s sign in the eponychium.

This case report has been prepared in accordance with the CARE guidelines. The completed CARE Checklist is provided as online supplementary material (for all online suppl. material, see https://doi.org/10.1159/cde/abvag017).

## Case Report/Case Presentation

A 33-year-old Saudi male noticed a progressive color change in his left little fingernail over 2 months. He reported no trauma, inflammation, drug intake, or systemic illness and had no family history of melanoma. On examination, a 3-mm wide longitudinal brownish-black band was seen on the left little finger covering the full length of the middle part of the nail plate with periungual extension of the pigmentation (positive Hutchinson sign) in a Fitzpatrick type IV patient. Dermoscopy revealed a solitary, light to dark gradient of brown-colored band with regularly spaced, sharp, parallel lines that showed varying thicknesses and brush-like extension into the eponychium and cuticle (Fig. 1, 2). A nail matrix tangential excisional biopsy of the pigmented proximal matrix lesion was performed (Fig. 3). Histopathology showed increased basal melanin with normal, bland-looking melanocytes, with no melanocytic nesting or an increase in their numbers, consistent with subungual melanosis (Fig. 4). An immunohistochemical stain was done to rule out any lentiginous growth or pagetoid spread patterns (Fig. 5). The patient was followed for 6 months. The nail completely regrew with no dystrophy, and the band had recurred.

**Fig. 1.**
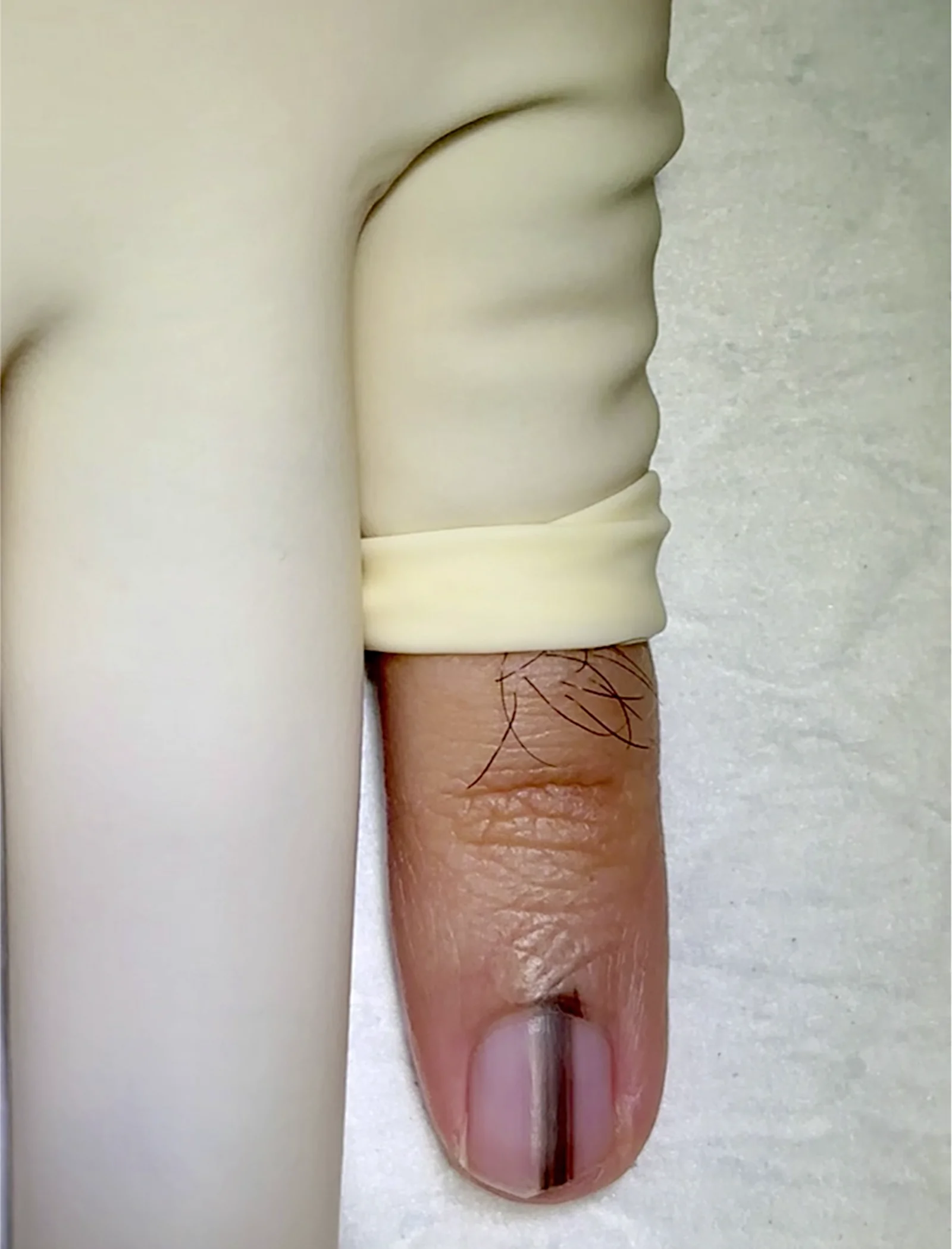
Clinical photograph of the left little fingernail, showing a 3-mm wide longitudinal brownish-black band with periungual extension of pigmentation into the eponychium (positive Hutchinson’s sign).

**Fig. 2.**
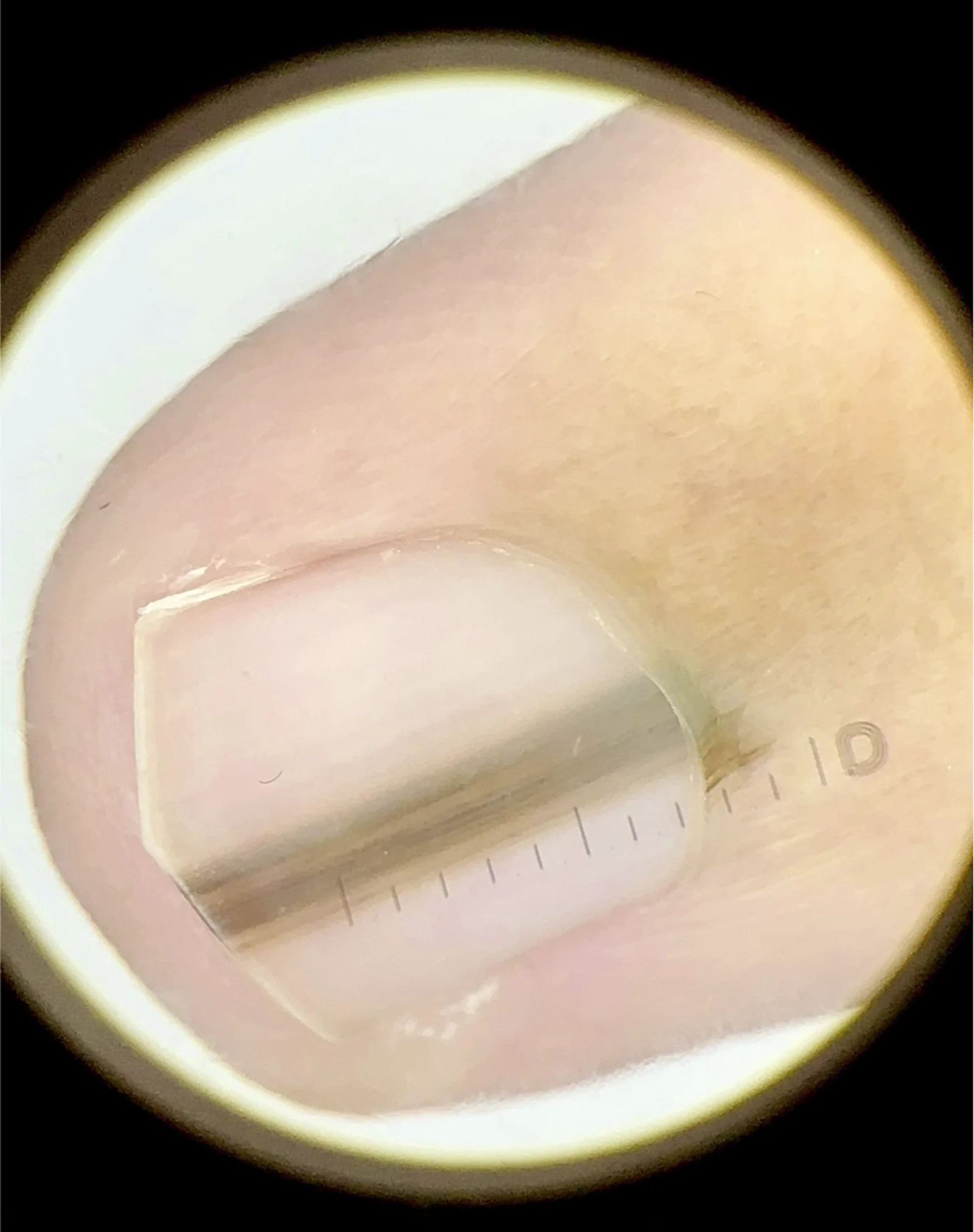
Dermoscopic image demonstrating a solitary light colored brown longitudinal band over a tan brown background, with irregularly spaced parallel lines of varying thickness and brush-like extension into the proximal nail fold.

**Fig. 3.**
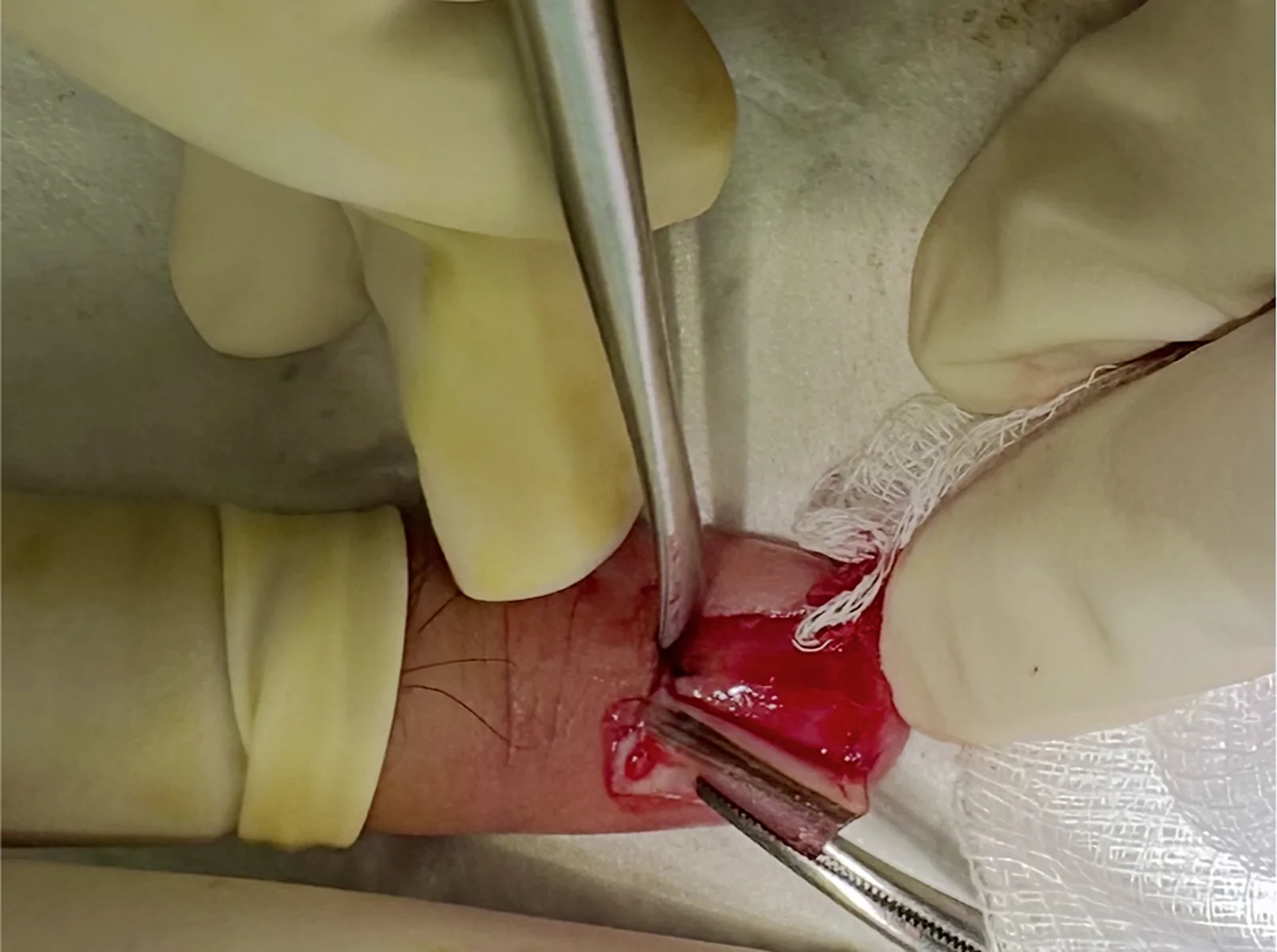
Intraoperative image after lateral reflection of the nail plate, showing a black macule located at the center of the proximal matrix epithelium (notice the pigmented band in the reflected plate).

**Fig. 4.**
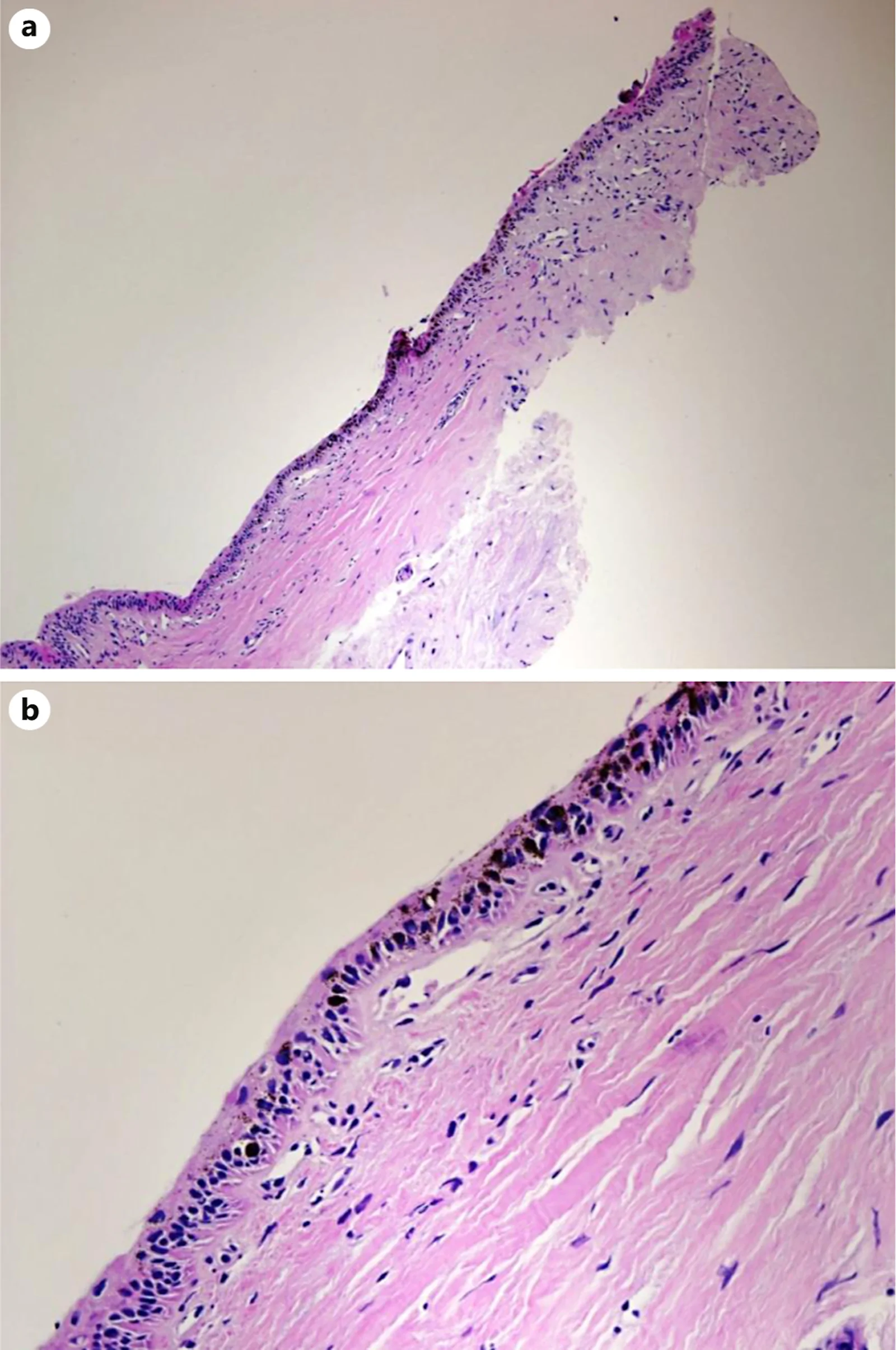
**a** Histopathology of the nail matrix specimen (H&E stain, ×40) showing increased basal layer melanin pigmentation of the nail matrix epithelium. **b** High power view (H&E ×200) demonstrates increased melanin pigment within the basal keratinocytes. Melanocytes appear morphologically bland, with no evidence of atypia, nesting, or pagetoid spread, consistent with subungual melanosis.

**Fig. 5.**
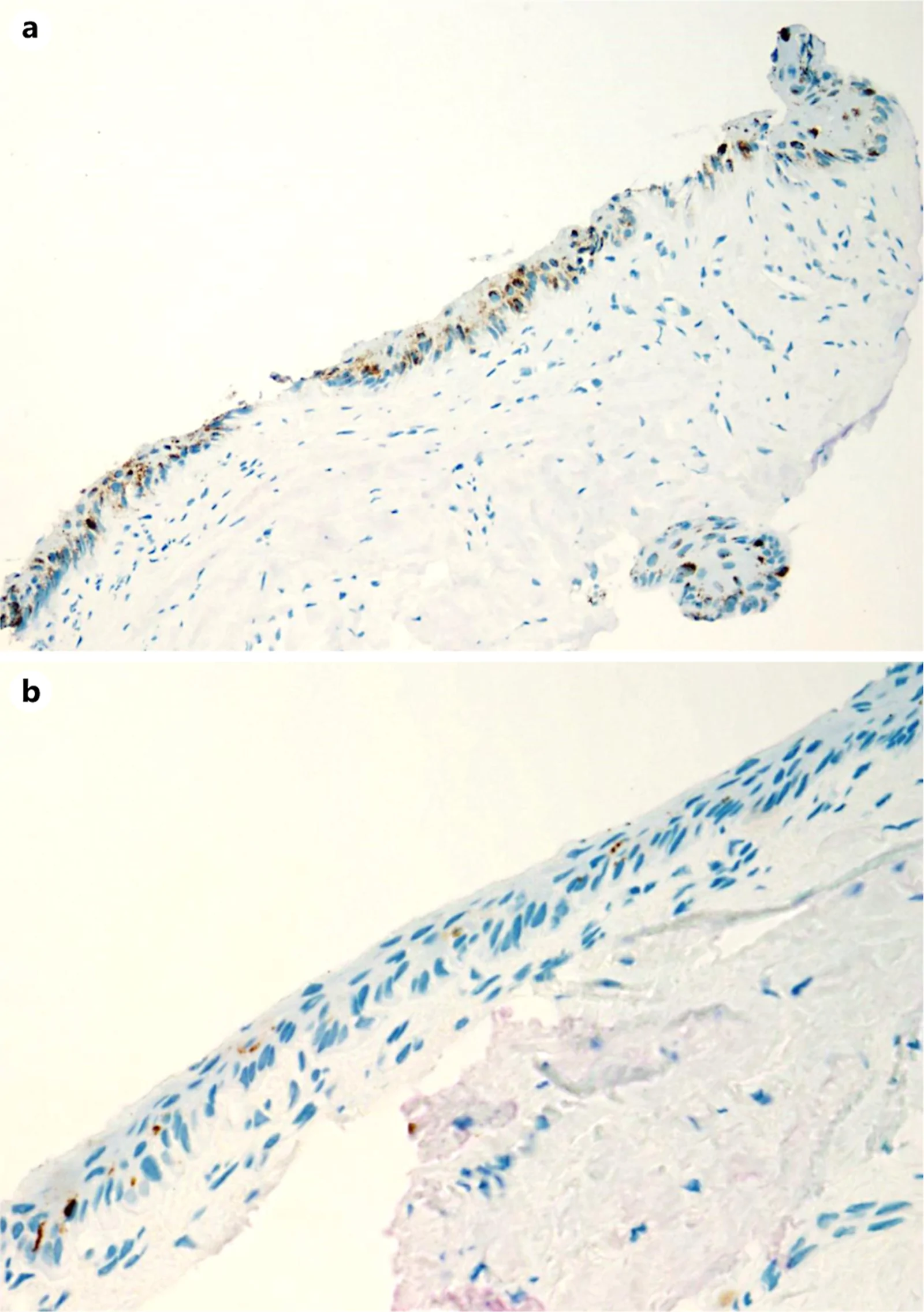
**a**, **b** High-power view (Melan-A immunostain) highlights a few scattered melanocytes along the nail bed epithelium with no lentiginous melanocytic proliferation or pagetoid spread.

## Discussion

Longitudinal melanonychia (LM) is characterized by a pigmented band, ranging from brown to black, that extends from the nail matrix to the free edge of the nail plate [5–7]. The ABCD rule for early detection of subungual melanoma is a useful tool to differentiate between benign and suspicious-looking melanonychia [8]. Our patient has essentially met several criteria warranting nail matrix biopsy, including adulthood, monodactylous, rapid progression with positive Hutchinson sign [3, 5, 6, 9]. A positive brushstroke Hutchinson sign has been described as a sign of pediatric hyponychium melanocytic nevi [10]; however, to our knowledge, adult presentation of benign brushstroke-like Hutchinson’s sign over the eponychium remain extremely rare, with only two previously reported cases [11]. Table 1, adapted from the existing literature and expanded with the current case, highlights the key differences between benign and malignant Hutchinson’s sign.

**Table 1.** Clinical and dermoscopic distinctions between benign and malignant Hutchinson’s sign

Feature	Benign causes (e.g., melanocytic nevus, subungual melanosis, ethnic pigmentation)	Malignant causes (subungual melanoma)
Age of onset	Younger patients, often stable	Middle-aged to older patients
Number of nails	Often multiple (especially in drug-induced or ethnic pigmentation)	Usually single nail (monodactylous)
Band width	Narrow (<3–4 mm), stable, or slowly progressive	Irregular, variegated (brown, black, gray, sometimes red)
Line pattern (dermoscopy)	Regular, parallel, evenly spaced	Irregular, disrupted, varying spacing and thicknesses
Extension onto nail folds (Hutchinson’s sign)	Rare, may appear as faint, brush-like, or uniform pigmentation	More intense, irregular periungual spread
Histopathology	Increased melanin, bland melanocytes, no atypia	Atypical melanocytes, nesting, pagetoid spread, dermal invasion
Outcome	Benign, non-progressive, no recurrence after biopsy	Progressive, invasive potential, requires wide excision

Nail matrix excisional biopsy is the gold standard for diagnosing atypical LM [12]. Nail matrix tangential excision with a 1 mm thick specimen is the preferred biopsy technique, where accurate diagnosis can be obtained without compromising the integrity of the growing nail [7, 9]. Biopsy of suspicious lesions remains essential, even when benign features are suspected.

Melanocytic activation, or subungual melanosis, is a term used to describe melanin overproduction from a normal number of matrix melanocytes and is functionally analogous to cutaneous ephelides (freckles). It is the leading cause of LM that typically affects multiple digits, most commonly the first three fingers, and is likely triggered by several reported factors [7, 13]. Unlike what has been reported in the literature, our patient had the melanocytic activation limited to a single digit, with no identifiable triggering factor.

## Conclusion

This case highlights that Hutchinson’s sign, although a red flag for subungual melanoma, may rarely occur in benign entities such as subungual melanosis. Comprehensive dermoscopic evaluation and confirmatory histopathology are essential to avoid both over- and under-treatment.

## Statement of Ethics

Ethical approval was not required for this study, in accordance with local and national guidelines, as this manuscript reports a single clinical case. Written informed consent was obtained from the patient for publication of this case report and any accompanying images.

## Conflict of Interest Statement

The manuscript has been read and approved by all authors, who have no conflicts of interest to declare.

## Funding Sources

This study was not supported by any sponsor or funding.

## Author Contributions

F.A. contributed to patient evaluation, clinical management, and manuscript drafting. A.A. and M.A. contributed to dermoscopic assessment, histopathological interpretation, and critical revision of the manuscript. All authors reviewed and approved the final version of the manuscript.

## Data Availability

All data generated or analyzed during this study are included in this article. Further inquiries can be directed to the corresponding author.
